# The $\gamma$ function in quantum theory II. Mathematical challenges and paradoxa

**DOI:** 10.1007/s10910-021-01311-w

**Published:** 2021-12-04

**Authors:** Zs. É. Mihálka, M. Nooijen, Á. Margócsy, Á. Szabados, P. R. Surján

**Affiliations:** 1 Department of Theoretical Chemistry, The University of Waterloo, Waterloo, Canada; 2 Institute of Chemistry, Laboratory of Theoretical Chemistry, ELTE Loránd Eötvös University, P.O.B. 32, Budapest 112, 1518 Hungary; 3 Hevesy György PhD School of Chemistry, ELTE, Budapest 112, Hungary

**Keywords:** Square root of Dirac-delta, Gamma function, Kinetic postulate

## Abstract

While the square root of Dirac’s $\delta$ is not defined in any standard mathematical formalism, postulating its existence with some further assumptions defines a generalized function called $\gamma (x)$ which permits a quasi-classical treatment of simple systems like the H atom or the 1D harmonic oscillator for which accurate quantum mechanical energies were previously reported. The so-defined $\gamma (x)$ is neither a traditional function nor a distribution, and it remains to be seen that any consistent mathematical approaches can be set up to deal with it rigorously. A straightforward use of $\gamma (x)$ generates several paradoxical situations which are collected here. The help of the scientific community is sought to resolve these paradoxa.

## Introduction

In a recent paper [1], hereafter referred to as paper I, the existence of a (generalized) function $\gamma (x)$ was postulated which satisfies the following axioms:

1a $$\begin{aligned}&\gamma (x) = 0 \quad \text {for} \quad x \ne 0 \end{aligned}$$

1b $$\begin{aligned}&\int \limits _{-\infty }^\infty \ \gamma ^2 (x) \ f(x) \ dx \ = \ f(0) \end{aligned}$$

1c $$\begin{aligned}&\int \limits _{-\infty }^\infty \ \gamma (x) \ f(x) \ \gamma ^{\prime \prime }(x) \ dx \ = \ 0 \end{aligned}$$

for any smooth, nonsingular function *f*(*x*), the prime indicating derivative. Axiom (1b) implies that $\int \ \gamma ^2 = 1,$ that is, function $\gamma$ is square-integrable. This, together with (1a) implies that $\gamma$ is singular at the origin. Axioms (1a–1b) identify $\gamma (x)$ as a square-root of the Dirac’s $\delta$, while (1c) was termed as the “kinetic postulate” for reasons given below.

With a trivial correction of Eqs. (1) indicating complex conjugates, function $\gamma$ (or wave functions constructed by it) can bear a complex phase factor. This, of course, would not affect any of the matrix elements discussed below. While even more complex functions could be considered then, in the present work we deal with real functions for simplicity.

The physical/chemical interpretation of the above axioms is as follows. A unit point charge clamped at the origin possesses the charge density

$$\begin{aligned} \rho (r) = \delta (r). \end{aligned}$$

If, in the spirit of a quasi-classical theory, one wants to associate a wave function to this charge density, one should formally write

$$\begin{aligned} \psi (r) = \sqrt{\delta (r)} = \gamma (r), \end{aligned}$$

which, however, is a non-existent object among either traditional functions or distributions. This does not generate any problem in quantum mechanics, as a static point charge (electron) is not legal there – it would contradict e.g. the Heisenberg uncertainty principle.

In the theory under discussion one’s aim is to elaborate a formalism which can deal with (quasi)classical objects using (a part of) the formalism of quantum mechanics: operators and expectation values. This is the motivation to search for a “wave function” of a resting charge, the latter being denoted here by $\gamma (r)$. Keeping this particle at rest requires to ensure that its kinetic energy is zero. This is satisfied by (in Cartesian coordinates and one dimension)

$$\begin{aligned} \langle {\hat{T}} \rangle = - \frac{\hbar ^2}{2m} \langle \gamma | \gamma ^{\prime \prime } \rangle \ = \ - \frac{\hbar ^2}{2m} \int \limits _{-\infty }^\infty \ \gamma (x) \ \gamma ^{\prime \prime } (x) \ dx \ = \ 0 \end{aligned}$$

as a special case of axiom (1c) for $f(x) = 1$.

The above equation clearly contradicts Heisenberg’s uncertainty relation. This is intentional, as the present aim is to develop a quasi-classical theory exhibiting classical features.

The three axioms above have been used in paper I for some examples, and the results recapitulated in Sect. 3 have been obtained. While the results listed there are noteworthy, the mathematical foundations of function $\gamma$ are still lacking. The aim of this paper is to collect all mathematical problems connected to function $\gamma$ that are known to us, in the hope that readers of this Journal can contribute to solving them. Apostrophing from Bernoulli: *“Problema novum, ad cuius solutionem mathematici invitantur”* (Joan Bernoulli, Opera Omnia, Tomus I.)

## Trivial properties of $\gamma (x)$

We work under the assumption that relations of elementary calculus, e.g., the chain rule or integration by parts, apply to expressions involving $\gamma$. If doing so, some properties of $\gamma (x)$ follow from axioms (1a–1b), that is, from the identification of $\gamma ^2(x)$ to $\delta (x)$. From the basic property of the latter,

2 $$\begin{aligned} \int \limits _{-\infty }^\infty \ f(x) \delta (x) \ dx = f(0), \end{aligned}$$

which is valid for any well-behaved function *f*(*x*), it follows (via integrating by parts) that

3 $$\begin{aligned} \int \limits _{-\infty }^\infty \ \delta ^{(n)}(x) \ f(x) \ dx \ = \ (-1)^n \ f^{(n)}(0) \end{aligned}$$

with the superscript (*n*) indicating *n*-th derivative. Properties of $\gamma$ arise by substituting $\gamma ^2(x)$ in place of $\delta (x)$. For the first derivative, e.g., one obtains:

4 $$\begin{aligned} \int \limits _{-\infty }^\infty \ \gamma (x) \ f(x) \gamma ^\prime (x) \ dx \ = \ - \ \frac{1}{2} \ f^\prime (0) \end{aligned}$$

which is a fundamental property of function $\gamma$. To obtain (4), one simply uses that $(\gamma ^2)^\prime \equiv 2 \ \gamma \ \gamma ^\prime$.

It is noteworthy that the value of the integrals of type $\int \gamma \gamma ^{\prime \prime }$, which are postulated to be zero by axiom (1c), can never be determined from these manipulations. This may give the impression that one is free to define this integral within the present formalism, and if this is true, one can apply definition (1c), in order to meet the physical interpretation of the kinetic integral at the quasiclassical level.

The properties given above are sufficient to treat the applications shown in paper I collected below.

## Summary of previous results

To improve the readability of this article, we recollect the basic results from paper I.

### The H atom *s* states

The H atom was described by the Ansatz

5 $$\begin{aligned} \varPsi _{ns} \ = \ {{\mathcal {N}}}_n \ r^{n-1} \ \gamma (r-r_n) \ Y_{00}, \ \ n=1,2,3,\cdots \end{aligned}$$

in spherical coordinates, where $Y_{00}=\frac{1}{\sqrt{4\pi }}$ is the normalized *s*-type spherical harmonic function. This is interpreted physically as a bubble model of the H atom, where the *s*-electron with principal quantum number *n* is distributed on the surface of a sphere of radius $r_n$. This means that the electron rests radially but it is delocalized angularly. The energy of the H atom was evaluated by standard quantum mechanical rules and using axioms (1a-1c). The result is:

6 $$\begin{aligned} E_{ns} (r_n)& = \ \langle \varPsi _{ns} | {\hat{H}}_{\text{ hydrogen }} | \varPsi _{ns} \rangle \ = \ T \ + \ V \nonumber \\ &= \frac{n^2}{2 \ r_n^2} - \ \frac{1}{r_n} \end{aligned}$$

in atomic units. It has minima wrt $r_n$ at $r_n = n^2$:

7 $$\begin{aligned} E_{ns} \ = \ - \frac{1}{2} \ \frac{1}{n^2}, \end{aligned}$$

i.e., the exact energies of the hydrogenic $|ns\rangle$ states were obtained. Note that after minimization the energy formula satisfies the virial theorem in the form $2T = -V$. Conversely, instead of minimization, the same energy formula (7) can be obtained by fixing $r_n$ in (6) to satisfy the virial theorem.

### The harmonic oscillator

Considering the standard Hamiltonian

$$\begin{aligned} {\hat{H}} \ = \ - \frac{1}{2} \ \frac{d^2}{dx^2} \ + \ \frac{1}{2} \ \omega ^2 \ x^2 \end{aligned}$$

(in atomic units and for unit mass) and the wave function Ansatz

$$\begin{aligned} \varPsi _n \ = \ {{\mathcal {N}}}_n \ x^n \ \big [ \gamma (x-x_n) \ + \ \gamma (x+x_n) \big ], \ \ n=0, 1, 2, \cdots , \end{aligned}$$

where $x_n$ are analogues of the classical turning points, the normalization constant was found to be

$$\begin{aligned} {{\mathcal {N}}}_n = \frac{1}{\sqrt{2} \ x_n^n}. \end{aligned}$$

The resulting energy formula is

8 $$\begin{aligned} E_n(x_n) \ = \ \frac{1}{2} \ \Big ( \frac{n^2}{x_n^2} \ + \ \omega ^2 \ x_n^2 \Big ) \end{aligned}$$

having the minimum wrt parameters $x_n$

$$\begin{aligned} E_n \ = \ n \omega , \end{aligned}$$

at $x_n \ =\ \sqrt{\frac{n}{\omega }}$, to be compared with the exact quantum mechanical spectrum $E_n = (n+\frac{1}{2}) \omega$. This result correctly gives back the quantized energies of the oscillator regarding the equidistant energy levels separated by $\omega$, but, as a consequence of the quasi-classical nature of the model wave function, does not provide the zero point energy $\frac{1}{2}\omega$.

We add here that the virial theorem for the harmonic potential requires $T = V$. It is satisfied by the above result after variation, which is easy to check upon substituting the optimal $x_n \ =\ \sqrt{n / \omega }$ into Eq. (8). Conversely, requiring that $T=V$ in (8) and solving for $x_n$, the same energy results emerge.

### The He atom

A rough model of the helium atom was constructed in which the two electrons are distributed on the surface of a sphere, occupying positions with maximum distance from each other (the “north-south” model). The ground state energy was –3.06 a.u., slightly below the exact quantum mechanical energy − 2.9. a.u. of He.

## The impossibility of the kinetic postulate

Although the kinetic postulate (1c) has been used in paper I. with success, here we show an argument indicating that it cannot be true for all *f*(*x*). Starting from

9 $$\begin{aligned} \gamma ^2(x) \ = \ \delta (x) \end{aligned}$$

and taking its second derivative one has:

10 $$\begin{aligned} 2 \ \gamma ^{\prime 2} \ + \ 2 \ \gamma \gamma ^{\prime \prime } \ = \ \delta ^{\prime \prime }. \end{aligned}$$

Multiplying this by *f*(*x*) and integrating yields

$$\begin{aligned} 2 \ \int \limits _{-\infty }^\infty \ f(x) \ \gamma ^{\prime 2} dx \ + \ 2 \ \int \limits _{-\infty }^\infty \ f(x) \ \gamma \gamma ^{\prime \prime } dx \ = \ f^{\prime \prime }(0), \end{aligned}$$

where property (3) of the $\delta$-function was used.

It is easy to see that this result leads to a contradiction for certain *f*(*x*). Consider a function which is positive everywhere, integrable and differentiable, and has a negative second derivative at the origin. An example is a gaussian. For this, the rhs is negative, while the first integral at the lhs is nonnegative. Therefore the second integral, which is just the kinetic postulate, cannot be zero for such an *f*(*x*).

There are, however, functions *f*(*x*) with other properties, for which the kinetic postulate holds, but as we see here, it cannot hold generally. This fact was not known to us when paper I was completed.

Accordingly, the situation is quite challenging: while (1c) is not true in general, its use as it was done in paper I and summarized here in Sect. 3, has lead to meaningful results.

The possible explanation of this paradox is currently being investigated in our laboratory and will be published in a forthcoming paper. The line of this investigation is motivated by the fact that a well-known origination of the Dirac’ $\delta$ is a limit of a valid family of functions (the so-called $\delta$-series). The above paradox makes this unlikely for $\gamma$, in connection to kinetic postulate. In a future paper we will pursue this approach; our preliminary results are encouraging.

At the present stage of research, to get rid of the contradiction among axioms (1) generated by requiring (1c) for any function *f*(*x*), we may modify this postulate to the weaker condition

$$\begin{aligned} \int \limits _{-\infty }^\infty \ x^k \ \gamma (x) \ \gamma ^{\prime \prime }(x) \ dx = 0, \end{aligned}$$

where *k* is a finite, nonnegative integer. This does not generate any contridiction to our current knowledge, but is sufficient to carry out all derivations reported in paper I.

##  The concept of singularity strength

An alternative introduction of the $\gamma$ function could be to consider the following properties:

11a $$\begin{aligned} \int \limits _{-\infty }^\infty \ \gamma (x) \ dx \,= & {} \ 0 \end{aligned}$$

11b $$\begin{aligned} \int \limits _{-\infty }^\infty \ \gamma ^2(x) \ dx \,= & {} \ 1 \end{aligned}$$

11c $$\begin{aligned} \int \limits _{-\infty }^\infty \ \gamma ^n(x) \ dx \,= & {} \ \infty \quad \text {for integer} \ n \ge 3, \end{aligned}$$

with $\gamma (x)$ being almost everywhere zero with the exception of the point $x=0$. Of these, (11b) matches (1b), the identification of $\gamma ^2$ to Dirac’s $\delta$. Eq. (11a) expresses that, while $\gamma (x)$ is singular at the origin as a consequence of (11b), it is *not singular enough* to yield a nonzero integral. Eq. (11c) indicates that $\gamma ^3$ and all higher powers of $\gamma$ are so singular at the origin that, in spite of being zero everywhere else, their integrals are divergent.

Accordingly, one may define the *singularity strength* $\frac{1}{\nu }$ of a function *g*(*x*) which is almost everywhere zero by defining $\nu$ as

12 $$\begin{aligned} \int \limits _{-\infty }^\infty \ g^\mu (x) \ dx \ = \ \left\{ \begin{array}{l} 0 , \ \text { if } \ 0< \mu < \nu \\ 1 , \ \text { if } \ \mu = \nu \\ \infty , \ \text { if } \ \mu > \nu , \end{array} \right. \end{aligned}$$

with $g^\mu$ indicating the $\mu$-th power of *g*(*x*). Note that in the case of $\mu =\nu$, the result can be any finite number which can be required to be one by appropriate normalization. With this definition, the singularity strength of Dirac’s $\delta$ is 1, while that of the $\gamma$ function equals $\frac{1}{2}$. Note that parameter $\nu$ is not necessarily an integer.

Given a smooth and bounded function *f*(*x*), Eqs.(11a)-(11b) can be generalized as

13a $$\begin{aligned} \int \limits _{-\infty }^\infty \ f(x) \ \gamma (x) \ dx \,= & {} \ 0 \end{aligned}$$

13b $$\begin{aligned} \int \limits _{-\infty }^\infty \ f(x) \ \gamma ^2(x) \ dx \,= & {} \ f(0). \end{aligned}$$

Since $\gamma (x)$ is zero everywhere for $x \ne 0$, function *f*(*x*) can affect integrals (13b) only through its finite value *f*(0).

### Remark

There is also an intuitive argument suggesting that Eq. (13a) may hold:

$$\begin{aligned} \int \limits _{-\infty }^\infty \ f(x) \ \gamma (x) \ dx \equiv \int \limits _{-\infty }^\infty \ \frac{f(x)}{\gamma (x)} \ \gamma ^2(x) \ dx = \frac{f(0)}{\gamma (0)} = 0. \end{aligned}$$

This equation is not precise from the mathematical point of view, since function $f(x)/\gamma (x)$ is neither smooth, nor bounded for $x \ne 0$. However, within the integral, it exhibits a removable singularity, since the numerator contains $\gamma ^2$. After integration, in course of which a generalization of Eq. (2) is used, the emerging function $f(0)/\gamma (0)$ is bounded and, $\gamma (0)$ being infinite, it is hard to assign to it any values other than zero.

## Comments on previous mathematical efforts

As known, a rigorous formulation of the Dirac-$\delta$ and similar generalized functions can be done within the theory of distributions [2, 3]. Motivated by the so called *impossibility theorem* of Schwartz [4], stating that no associative multiplication may exist among distributions, several ideas have been studied to deal with multiplication of generalized functions. Some authors have also addressed the question of the square-root of the Dirac-$\delta$. A few of these theories are listed below. Our conclusion from this list is neither of these previous efforts solves the question of the existence of $\gamma (x)$ as defined by axioms (1).

- **Colombeau algebra**
  The Colombeau algebra [5, 6] is a structure obtained by taking the quotient of an algebra with respect to an ideal within it. Distributions are considered to be elements of this algebra via an embedding, thus their multiplication can be defined. Not all elements of a Colombeau algebra correspond to distributions. The association of elements is defined so that effect of associated elements (denoted as $\approx$) on test functions differs by an infinitesimal number. Thus the concept of infinitesimals (and consequently, generalized numbers) [7] is connected to Colombeau’s theory of generalized functions. The Colombeau algebra generalizes pointwise multiplication of classical functions. However, product of two classical functions in the Colombeau algebra is not equal to their classical product, ‘only’ associated to it. This allows to e.g., construct for any complex number *c* a generalized function *g* that fulfills $g^2\approx c \cdot \delta$, i.e., a square root of Dirac’s delta in some sense (Ref. [8] Example 10.6.). Apparently these constructions do not conform to the kinetic postulate (1c) (i.e., $g\cdot g'' \approx 0$ does not hold).
- **Thurber’s theory** [9]
  Thurber also utilizes the concept of ‘infinitesimal’ and ‘infinitely large’ quantities (generalized numbers) appearing in the context of non-standard analysis [7], and defines fractional powers of delta via the function $d(x)=c n^{1/2} \exp (-nx^2)$, where *n* is infinitely large. Thurber and Katz perform calculations with $d^p(x)$ e.g., on
  - the self energy *U* of a classical electron,
  - non-standard wave packets.
  In the former case, they obtain $U \sim n^b$, where *b* depends on *p*, and choose parameter *p* such that $b=0$ and *U* is finite.
- **Craven’s formalism** [10]
  Craven also relies on the concept of infinitesimals and generalized functions to obtain a square root of $\delta$, but observations similar to those made above in connection with Ref. [8] hold.
- **Hanzon’s theory** [11]
  Hanzon treated the distribution equation $f^2=\delta$ either on a unit circle of the complex plane or on the real axis, in the latter case considering a periodic $\delta$ function
  $$\begin{aligned} \sum \limits _{k=-\infty }^\infty \delta (x-k). \end{aligned}$$
  Neither is the case here, and we note also that his definition of multiplication of distributions makes use the concept of convolution, that we do not use here either.

## Open questions and unusual properties of $\gamma (x)$

We proceed now to collect some paradoxical properties of $\gamma (x)$, in addition to the one discussed above in Sect. 4. We emphasize that we cannot resolve all of these paradoxa, but the physical information provided by the use of $\gamma (x)$, i.e., the successful applications presented in paper I, suggests that *there should exist* such a resolution, maybe within a mathematical framework yet unexplored.

In this Section, we address the following particulars:

- Function $\gamma$ is unexpandable in a separable basis of $L^2$
- Violation of some standard quantum mechanical theorems
- Linear combination of $\gamma$-containing terms
- The question of the closure relation
- Some functions containing $\gamma (x)$ form a zero-length subset
- Blockdiagonality of Hamiltonians
- Full support of the zero differential overlap approximation by $\gamma$-functions

###  Function $\varvec{\gamma }$ is unexpandable in a separable basis of $L^2$

Let us study first, for comparison, the expansion of the Dirac $\delta$. Let an orthonormal basis in the $L^2$ function space be formed by real functions $\chi _k(x)$. Then the expansion writes:

$$\begin{aligned} \delta (x) = \sum _k \ c_k \ \chi _k(x) \end{aligned}$$

where the expansion coefficients emerge by evaluating the scalar products

$$\begin{aligned} c_k \ = \ \langle \chi _k | \delta \rangle \ = \ \int \limits _{-\infty }^\infty \ \chi _k(x) \ \delta (x) \ dx \ = \ \chi _k(0). \end{aligned}$$

The series thus obtained,

$$\begin{aligned} \delta (x) = \sum _k \ \chi _k(0) \ \chi _k(x) \end{aligned}$$

is simply the special case of the completeness relation $\delta (x-y) = \sum _k \ \chi _k(y) \chi _k(x),$ and is clearly divergent since $\delta (x) \notin L^2$, as manifested by $\sum _k \ \chi _k(0)^2 \ = \ \infty .$ This means that the Dirac’s $\delta$, although not square-integrable, can be regarded as a limit of $L^2$ functions (the limit taken point wise, not with respect to norm).

How does the above modify if using $\gamma (x)$ in place of $\delta (x)$? Writing

$$\begin{aligned} \gamma (x) = \sum _k \ c_k \ \chi _k(x) \end{aligned}$$

and expressing the expansion coefficients one gets:

$$\begin{aligned} c_k \ = \ \langle \chi _k | \gamma \rangle \ = \ \int \limits _{-\infty }^\infty \ \chi _k(x) \ \gamma (x) \ dx \ = \ 0, \end{aligned}$$

since $\chi _k$-s are nonsingular and the singularity strength (cf. Sect. 5) of $\gamma$ is $\frac{1}{2}$. Accordingly, $\gamma (x)$ is represented by a sum of an infinite number of zeros. This function has therefore no practical expansion. Strictly speaking, it is not an element of $L^2$, as it cannot be considered as an accumulation point of any sequence in $L^2$. Thus we see the paradoxical situation that while $\gamma (x)$ is square-integrable, it is not an element of the $L^2$ function space. Here we merely pose the question whether it is possible to extend the concept of the $L^2$ space so that the extended space contains function $\gamma (x)$ and the functions derived from it.

###  Consequences of Sect. 7.1: violation of standard theorems

Some fundamental theorems of quantum mechanics, like the variational theorem (the upper bound nature of the energies of trial functions) or the Eckart theorem (on the convergence of trial wave functions) are proven by utilizing that any trial function is expandable in the complete space of exact eigenstates [12]. Section 7.1 above has the message that function $\gamma$ is not expandable. Therefore, when evaluating an energy as an expectation value of a wave function constructed by $\gamma (x)$ it may happen that we get an energy which is lower than the exact ground state (by violating the variational theorem), or we may get the exact energy while our wave function is not exact (violating the Eckart theorem). This latter exactly happens in the case of the hydrogen atom.

### Linear combination of $\varvec{\gamma }$-containing terms

Consider a set of functions $\{b_k\}$ defined as

$$\begin{aligned} b_k(x) = {{\mathcal {N}}}_k \ x^k \ \gamma (x-x_0) \end{aligned}$$

with $x_0$ (the center of function $\gamma$) fixed. The normalization factor, using condition $\langle b_k | b_k \rangle = 1$, evaluates to

$$\begin{aligned} {{\mathcal {N}}}_k \ = \ \frac{1}{x_0^k}. \end{aligned}$$

It may be tempting to expand wave functions in terms of $b_k(x)$ instead of using an intuitively selected wave function Ansatz as it was done e.g. in paper I. However, when checking the full overlap matrix, one finds that

$$\begin{aligned} S_{kl} \,= & {} \ \langle b_k | b_l \rangle \\ \,= & {} \ {{\mathcal {N}}}_k \ {{\mathcal {N}}}_l \ \int \limits _{-\infty }^\infty \ x^k \gamma (x-x_0) \ x^l \gamma (x-x_0) \ dx \\ \,= & {} \ \frac{x_0^{k+l}}{x_0^k \ x_0^l} = 1, \end{aligned}$$

thus such functions form a redundant set which makes them inappropriate to form a basis. The resolution of this paradox is that $\gamma (x-x_0) = 0$ almost everywhere, namely it is zero everywhere with the exception of the point $x=x_0$. Therefore, one may write

$$\begin{aligned} b_k (x)= & {} {{\mathcal {N}}}_k \ x^k \ \gamma (x-x_0) = \underbrace{ {{\mathcal {N}}}_k \ x_0^k }_{1} \ \gamma (x-x_0) \\= & {} \gamma (x-x_0) \end{aligned}$$

for every integer *k*. The conclusion is that new basis functions cannot be generated by multiplying the same $\gamma$ function by different power functions. (Note that in paper I we used the Ansatz (5) for the hydrogenic *ns* states, but there the power functions multiply *different* $\gamma (r-r_n)$-s. A similar remark applies for the different states of the oscillator.) The above argument holds only if no derivatives of $b_k$ are considered, i.e., when $b_k$ enters a matrix element of a multiplicative operator. Thus, albeit $\langle b_k | b_l \rangle = 1$ even for $k \ne l,$ the kinetic energy matrix elements evaluated by these two functions will not be the same, generating another paradoxical propery of function $\gamma$.

###  The question of the closure relation

Let us investigate now the question whether functions $\gamma (r-\tau )$ for all $\tau$ form a basis in some sense, i.e., whether they satisfy some form of the completeness (closure) relation. Let us recapitulate first the similar property of the Dirac’s delta function. Instead of satisfying the discrete closure relation $\sum _k \psi _k(x) \psi _k(y) \ = \ \delta (x-y),$ which the discrete basis functions should obey in order to form a complete basis, the Dirac-$\delta$ functions at various positions $\tau$ satisfy the continuous closure relation

$$\begin{aligned} \int \limits _{-\infty }^\infty \ \delta (x-\tau ) \ \delta (y-\tau ) \ d\tau \ = \ \delta (x-y). \end{aligned}$$

This follows simply from the basic property of the Dirac-$\delta$, Eq. (2).

In comparison, when evaluating a similar integral for the $\gamma$ functions, one obtains:

14 $$\begin{aligned}&\int \limits _{-\infty }^\infty \gamma (x-\tau ) \ \gamma (y-\tau ) \ d\tau \nonumber \\&\quad = \left\{ \begin{array}{ll} 0 \, &{} \quad \text {if} \ x \ne y \\ \int \limits _{-\infty }^\infty \ \gamma ^2(x-\tau ) \ d\tau \ = \ 1 &{} \quad \text {if} \ x = y \end{array} \right. \nonumber \\&\quad = \delta _{x,y} \end{aligned}$$

with a somewhat uncommon notation for the Kronecker delta-symbol, which is typically used for discrete indices. Relation (14) suggest that functions $\gamma (x-\tau )$ for all $\tau$ satisfy a closure relation apart from normalization.

### On a zero-length subset

In his lecture notes on linear algebra [13], Löwdin discussed the case of nonzero vectors having zero norms. These originated form an indefinite metric of the space. Here we call the attention to the fact that some nonzero functions, derived from $\gamma (x)$, can have zero length as a consequence of the singular properties of $\gamma$.

A simple example is the function $f(x) = x\cdot \gamma (x)$. One could (wrongly) argue that this function is identically zero as *x* is zero at the origin while $\gamma (x)$ is zero everywhere else. The error is in forgetting that $\gamma$ is singular at the origin. To point out that this argument is indeed misleading, consider the derivative of *f*(*x*):

15 $$\begin{aligned} f^\prime (x) \ = \ \gamma (x) \ + \ x \cdot \gamma ^\prime (x) \ \ne 0. \end{aligned}$$

If *f*(*x*) were identically zero, its derivative would be the same, while $f^\prime (x)$ is apparently nonzero. On the contrary, it has a nonzero overlap with $\gamma (x)$:

$$\begin{aligned} \langle \gamma (x) | f^\prime (x) \rangle = \underbrace{ \langle \gamma (x) | \gamma (x)\rangle }_{1} + \underbrace{ \langle \gamma (x) | x \gamma ^\prime (x) \rangle }_{-\frac{1}{2}} \ = \ \frac{1}{2}, \end{aligned}$$

where Eq.(15) was substituted and properties (1b) and (4) were used. However, evaluating the square norm one finds:

$$\begin{aligned} || f ||^2 \ = \ \langle x \gamma | x \gamma \rangle \ = \ \int \limits _{-\infty }^\infty \ x^2 \ \gamma ^2(x) \ dx = 0, \end{aligned}$$

as a consequence of the trivial property of $\gamma ^2 = \delta$.

Accordingly, the nonzero function $f(x) = x\cdot \gamma (x)$ has a zero norm, it is an element of the zero length subspace of an extension of the $L^2$ space containing function $\gamma$ and functions emerging from it.

#### Remark

The example of $x\gamma (x)$ is not a rare one. It is easy to see that $g(x) \gamma (x)$ has a zero norm for any *g*(*x*) which is zero at the origin.

###  Blockdiagonality of Hamiltonians

This point will be shown first on the example of the H atom. Considering the functions given by Eq.(5) as a basis subset, one may represent the Hamiltonian of the H atom in this basis. The diagonal elements are given in Eq.(7). As to the off-diagonal elements $\langle \varPsi _{ms} | {\hat{H}} | \varPsi _{ns} \rangle$ for $m \ne n$, one observes that the corresponding integrals contain the products of $\gamma (r-r_m)$ and $\gamma (r-r_n)$ or derivatives thereof, and since $r_m \ne r_n$, these integrals vanish (see the discussion in point 7.7. below).

We have thus the unusual situation that, while the basis functions $\varPsi _{ns}$ are not exact eigenstates of the hydrogenic Hamiltonian, the latter is diagonal in this subset of basis functions with exact eigenenergies.

This situation is not characteristic to the H atom. Any “local” operators, i.e., those not affecting the place $x_i$ of singularity of $\gamma (x-x_i)$, show this feature, as well as terms of a Hamiltonian: the kinetic energy operator and the potential. This is due to the fact that functions $\gamma (x-x_i)$ with different centers $x_i$ manifest the full ZDO (zero differential overlap) model. This point will be discussed below in more detail.

###  Full support of ZDO approximation

This problem occurs when trying to treat two or more electrons.

The ZDO approximation played a central role in early days of quantum chemistry, when no ab initio computations were available for real chemical systems. Semiempirical theories applied the ZDO approximation as a tool of handling two-electron integrals [14–18]. The success of semiempirical methods motivated theoreticians to search some explanation why these work, in spite of the fact that ZDO treatment of two-electron integrals could not be justified numerically.

An interesting argument was emphasized by Fischer-Hjalmars [19]. Since in semiempirical theories the basis functions (typically AOs) are never explicitly specified when setting up the list of two-electron integrals, one can imagine that the original, overlapping AO basis set has been Löwdin-orthogonalized tacitly. It was indeed shown that the ZDO approximation is much less drastic in a Löwdin AO basis.

The set of $\gamma$ functions centered at different places is obviously an orthogonal one:

$$\begin{aligned} \langle \gamma (x-x_i) | \gamma (x-x_k) \rangle \ = \ 0 \qquad i \ne k, \end{aligned}$$

since the bra and the ket functions have no common point where they both differ from zero, and the singularity strength of $\gamma$-functions is $\frac{1}{2}$.

Recall that orthogonality of two spatial functions may occur from two rather different reasons. In the first case they share their nonzero measure domain, at least a part of it, but their nodal structure makes them orthogonal. In this case they are orthogonal only after integration, but their differential overlap is nonzero. The other case is when there is no point or domain where the two functions are simultaneously nonzero. These are the functions which fully satisfy the ZDO condition. To our knowledge, such functions were only imagined so far, but have never been explicitly constructed, apart from large-exponent gaussians located at remote places (Löwdin orthogonalization yields only an approximate ZDO). The use of $\gamma$ functions offers an explicit realization of ZDO basis sets.

There is a problem, however, which was present already in semiempirical quantum chemistry, but was somehow always swept under the rug: the role of ZDO in one-electron integrals. Namely, the ZDO condition was never used there, otherwise no off-diagonal elements would have been emerged, and no hopping integrals would have survived, i.e., no chemical bonds would have occurred. A partial explanation was that kinetic energy integrals do not contain differential overlap of the bra and the ket functions, since the ket is differentiated by the Laplacian. This argument, however, does not explain why not to use ZDO in one-electron potential integrals, like the nuclear-electron attraction, which was never applied either. Rather, these integrals were empirically approximated, often using the integral overlap of the bra and the ket in an empirical (Wolfsberg-Helmholtz) formula [20]. Such parametrization has led to much success even when neglecting two-electron interaction entirely, such as in Hoffmann’s seminal extended Hückel theory [21].

Further research has to be conducted to see whether functions $\gamma$ can be, in some manner, used in developing quasiclassical models for many-electron wave functions.

## Summary

This paper collects several striking properties of function $\gamma (x)$ introduced previously and associated to the square-root of Dirac’s $\delta$. We showed that the “kinetic postulate” (1c) cannot be true for arbitrary *f*(*x*), nevertheless, its use yields meaningful results detailed in paper I and excerpted in Sect. 3. A new concept, the singularity strength of a function which is zero almost everywhere, was introduced in Sec. 5. Finally, we showed that $\gamma (x)$

- is not expandable in $L^2$, thus it may violate standard quantum mechanical theorems
- functions $x^k \gamma (x-x_0)$ have unit overlap with $\gamma (x-x_0)$ after normalization
- functions $\gamma (x-y)$ satisfy a special form of the closure relation
- functions $g(x) \gamma (x)$ with $g(0)=0$ form a zero-length subset
- supports a full ZDO approximation.

Two main issues require further studies:

(A) How is it possible that in spite of the several paradoxical situations it exhibits, and especially in spite of the violation of the kinetic postulate, function $\gamma (x)$ leads to useful applications, including some exact results?
(B) Can functions constructed from $\gamma (x)$ be used to provide approximate description of many-electron systems in a quasi-classical way?

While continuing our research towards these directions, we shall be happy to receive any help from the scientific community in the above matters.
